# Factors influencing the outcome of cochlear implantation: what role is played by secondary and post-secondary education?

**DOI:** 10.1007/s00106-025-01647-8

**Published:** 2025-09-11

**Authors:** Christoph Broeder, Uwe Baumann

**Affiliations:** Department of Otolaryngology, Goethe University Frankfurt, University Hospital Frankfurt a. M., Theodor-Stern-Kai 7, 14, 60590 Frankfurt am Main, Germany

**Keywords:** Educational attainment, School degree, Cognition, Freiburg monosyllables, Oldenburg sentence test, Bildungsgrad, Schulabschluss, Kognition, Freiburger Einsilber, Oldenburger Satztest

## Abstract

**Background:**

Individual cognitive abilities are increasingly discussed as a potential factor influencing the outcomes of cochlear implant (CI) treatment. In this context, the present study investigated a possible correlation between the secondary and post-secondary education of a large cohort of CI recipients and their speech comprehension. Other variables with a potential influence on the treatment outcome included age at implantation, the duration of hearing loss, and the treatment mode.

**Methods:**

The study included data of *n* = 326 patients from the audiology database of the Frankfurt University ENT Clinic. Secondary and post-secondary education histories were collected as part of the anamnesis using a questionnaire. Speech comprehension was assessed using the Freiburg monosyllabic test (FBE) and the Oldenburg sentence test in noise (OLSA) at 6 and 12 months after implantation and correlated with various biographical and audiological factors.

**Results:**

Patients with higher educational qualifications showed better outcomes in the FBE at 6 months (ANOVA_Welch_; *F* (2, 104) = 5.60; *p* = 0.05) and at 12 months (*F* (2, 223) = 3.07; *p* = 0.05; *η*^*2*^ = 0.03) compared to those with vocational qualifications. In the speech comprehension in noise (OLSA), a significant group difference was observed 12 months postoperatively. Patients without qualifications or with other qualifications scored lower than those with vocational or higher qualifications (*F* (2, 74) = 4.41; *p* = 0.02; *η*^*2*^ = 0.11). Other significant factors influencing speech comprehension included the age at implantation and mode of care.

**Conclusion:**

The correlation between secondary and post-secondary education and speech comprehension after CI treatment was not unequivocal. Only the choice of post-secondary education showed a significant correlation with speech comprehension. Further research is required to confirm a potential relationship.

**Supplementary Information:**

The online version of this article (10.1007/s00106-025-01647-8) contains supplementary material.

## Abstract (de)

**Hintergrund:**

Individuelle kognitive Fähigkeiten werden in zunehmendem Maße als ein möglicher Faktor diskutiert, der das Ergebnis der Cochleaimplantat(CI)-Versorgung beeinflussen kann. Vor diesem Hintergrund wurde in der vorliegenden Studie ein möglicher Zusammenhang zwischen dem Schulabschluss und der Berufsausbildung einer größeren Gruppe von CI-Trägern und dem Sprachverstehen untersucht. Als weitere Variablen mit möglichem Einfluss auf das Versorgungsergebnis wurden das Alter bei Implantation, die Dauer der Hörminderung und der Versorgungsmodus einbezogen.

**Methodik:**

Insgesamt wurden *n* = 326 Patientendaten aus der Audiologie-Datenbank der HNO-Universitätsklinik Frankfurt in die Studie einbezogen. Bei diesen Patienten wurde die schulische und berufliche Laufbahn im Rahmen der Anamnese mittels Fragebogen erhoben. Das Sprachverstehen wurde mit dem Freiburger Einsilbertest (FBE) und dem Oldenburger-Satztest im Störgeräusch (OLSA) nach 6 und 12 Monaten bestimmt und mit verschiedenen biografischen und audiologischen Einflussfaktoren korreliert.

**Ergebnis:**

Fälle mit einem höheren Ausbildungsabschluss wiesen 6 und 12 Monate postoperativ ein besseres Ergebnis im FBE 6 M (ANOVA_Welch_; *F* (2, 104) = 5,60; *p* = 0,05) und FBE 12 M (*F* (2, 223) = 3,07; *p* = 0,05; *η*^*2*^ = 0,03) auf als Fälle mit einem beruflich-schulischen Abschluss. Auch beim Sprachverstehen im Störgeräusch (OLSA) zeigte sich 12 Monate postoperativ ein signifikanter Gruppenunterschied. Fälle ohne Abschluss/mit sonstigem Abschluss erzielten schlechtere Testergebnisse als solche mit einem beruflich-schulischen oder einem höheren Abschluss (*F* (2, 74) = 4,41; *p* = 0,02; *η*^*2*^ = 0,11). Andere signifikante Einflussfaktoren auf das Sprachverstehen waren das Alter bei der Implantation und der Versorgungsmodus.

**Schlussfolgerung:**

Die Korrelation zwischen dem schulischen und beruflichen Werdegang und dem Sprachverständnis nach CI-Versorgung war nicht eindeutig. Lediglich der Ausbildungsabschluss zeigte einen signifikanten Zusammenhang mit dem Sprachverstehen. Weitere Forschung ist erforderlich, um einen möglichen Einfluss des Bildungsgrades auf das Sprachverstehen zu bestätigen.

In the current literature, a large part of the variance in speech perception after cochlear implant (CI) fitting remains unexplained. Individual cognitive abilities as a probable influencing factor on CI outcome are currently the subject of debate. Part of this variance could be explained by the level of education as a potential indicator of patients’ cognitive abilities. Previous studies have not shown any significant correlation between the level of education and speech comprehension, but they highlight the necessity for further research.

The cochlear implant (CI) is a hearing prosthesis for the deaf and severely hearing impaired for whom a hearing aid is no longer sufficient. It is considered the standard therapy for patients with severe hearing loss. [21, 26]. As described by Lenarz et al., the outcome of CIs is highly individual and influenced by various factors. Etiology, duration, and onset of hearing loss as well as age at implantation are well-documented influencing factors in the literature [1, 3, 8, 22]. Not only do CIs improve speech perception and auditory comprehension but they also enhance the quality of life of their users [36, 39]. Continuous advancements in implant technology, CI processors, electrode designs, and software, along with changes in clinical care conditions, could lead to a change in the impact of these factors on speech perception. In two multicenter studies (1996, 2013), Blamey et al. showed a decline in the significance of biographical and audiological factors in predicting speech comprehension. In 2013, these factors accounted for only 10% of variance, compared to 21% in 1996 [2, 3]. More recent studies have continued to evaluate the relevance of well-established predictors. In a meta-analysis, Zhao et al. (2020) found that while preoperatively measured patient-specific factors significantly impact speech comprehension, they collectively explain only about 10% of the variance, thus offering limited support for clinical decision-making regarding CI treatment [41]. The predictability of CI success and the associated treatment planning remain limited overall. Studies suggest that older adults with hearing loss exhibit poorer speech comprehension than younger adults, despite similar results in pure-tone audiometry [6, 40]. This discrepancy may be related to age-related decline in cognitive functions such as processing speed, working memory, and selective attention. In quiet environments, speech processing occurs almost automatically, whereas in noisy settings, especially for individuals with impaired hearing, incoming neural activity patterns may be distorted, making it difficult to match them to stored representations [27]. These distortions demand cognitive resources for speech perception, leading to challenges for individuals with reduced cognitive processing speed. The speech processing model indicates that a significant portion of the variance in speech perception among older adults can be attributed to differences in cognitive performance [12]. Cognition is defined as an internal process involved in understanding the environment and determining appropriate actions [9]. Studies by Heydebrand et al. (2007) and Moberly et al. (2018) have examined cognitive abilities, such as auditory working memory, as potential influencing factors in CI outcomes, yielding disparate results [19, 32]. Heydebrand et al. reported no correlation between cognitive parameters and CI outcomes in a study of 37 participants at 6 months postoperatively [19]. By contrast, Moberly et al. showed that the results of speech comprehension correlate with auditory working memory in 31 patients [32]. Both authors concluded that further research is needed to determine the influence of cognitive effects on speech comprehension.

The present study investigated patients’ education level as an indicator of their cognitive abilities. The level of education includes the type of institution attended and the highest degree achieved by the patients. Numerous studies indicate that in normal-hearing individuals, higher education levels correlate with better cognitive performance [7, 30, 33]. In a review by Lövdén et al., the authors describe a positive relationship between education level and cognition. Similarly, Chen et al. found a significant association between higher education levels and greater overall cognitive performance in a sample of 659 normal-hearing older adults. Moreover, these individuals exhibited a delayed age-related decline in cognitive function [7]. It can be assumed that the described effects of education level on cognition also apply to individuals with hearing impairment. Therefore, education level may serve as an indicator of patients’ cognitive abilities. However, the relationship between education level and speech perception following CI treatment has received limited attention in the literature. Heutink et al. and Lazard et al. investigated the influence of education level on speech perception in cohorts of 129 and 2251 CI recipients, respectively [18, 25]. Neither working group was able to establish a significant correlation with the CI outcome. It is possible that the chosen sample sizes were too small or that the classification of educational levels was too broad, potentially obscuring significant correlations.

A clear definition of “CI outcome” is essential for establishing a potential correlation. In this study, CI outcome or CI success is defined as speech perception after CI fitting, measured using the Freiburg Monosyllabic Test (FMS) and the Oldenburg Sentence Test (OLSA). Other factors such as social participation, quality of life, and communication, which are generally considered part of CI outcomes, were not included. The present study aimed to investigate whether patients’ education level correlates with speech perception following cochlear implantation.

## Material and methods

### Data collection

The study involved a retrospective data collection of patients who received a hearing implant between October 2008 and October 2021. The datasets were retrieved from the audiology database of the Department of Otorhinolaryngology, University Hospital Frankfurt (ENT-Statistics Version: 4.1.522.546, Innoforce, Liechtenstein). Between 2017 and 2021, as part of the anamnesis, patients who underwent implantation within the specified period were asked to provide information about their educational and professional background. The responses were recorded in a designated database form (“Work and Life”). A database query generated an Excel spreadsheet (Version: 16.74; Microsoft Corp., Redmond, WA, USA) containing patient demographic data, treatment information, patient history, type of implantation, details on living circumstances, and entries from the “Work and Life” form. The database query for the period between October 2008 and October 2021 yielded 821 entries for patients who received CIs, Bonebridge implants (Med-EL, Innsbruck, Austria), or Vibrant Soundbridge implants (VSB, Med-EL, Innsbruck, Austria). Among these cases, 609 questionnaires regarding educational attainment (“Work and Life” form) were available. After applying additional inclusion criteria—native German speakers (resulting in 235 dropouts due to a different native language) and information on the mode of implantation (23 drop-outs due to missing data)—the study population was reduced to 351 remaining cases. For 120 cases with bilateral CI, data from the CI side with the better postoperative result in the FMS and lower signal-to-noise ratio (SNR) values in the OLSA were included in the study. In cases with discrepant test results, the average test performance across the follow-up intervals (6 and 12 months postoperatively) was calculated to determine the better-performing side. This approach prevented bilateral CI patients from contributing duplicate datasets regarding educational attainment, which could have led to data distortion. Additionally, cases were excluded if FMS results were unavailable (*n* = 6) or if auditory nerve defects were diagnosed (*n* = 2). After excluding patients with Bonebridge and Vibrant Soundbridge implants (*n* = 17), the final study population comprised 326 cases.

### Speech perception in quiet and noise

The study population included 190 female and 136 male patients (Table 1). The average age at implantation was 53.0 years (SD = 16.5). Under best-fitted hearing aid conditions, the preoperative mean score in the FMS was 16.5% (SD = 21.9). At 6 months postoperatively, the mean score improved to 57.4% (SD = 26.1), and at 12 months, it reached 63.9% (SD = 23.0). The speech reception threshold in noise (OLSA) improved by 0.6 dB SNR, decreasing from L50 = −0.8 dB SNR (SD = 2.5) at 6 months postoperatively to L50 = −1.4 dB SNR (SD = 2.2) at 12 months postoperatively. Following listwise case exclusion, 226 cases remained for correlation analysis with the FMS results, while 77 cases were included for correlation analysis with OLSA outcomes.

**Table 1:** Demographic data and speech perception results

	*N*	Minimum	Maximum	Mean	SD
Age in years	326	20.0	92.0	58.6	16.0
Age at implantation in years	326	2.2	88.4	53.0	16.5
FMS pre-OP (%)	217	0.0	85.0	16.5	21.9
FMS 6 M post-OP (%)	217	0.0	100.0	57.4	26.1
FMS 12 M post-OP (%)	217	5.0	100.0	63.9	23.0
OLSA 6 M (dB SNR)	77	−5.3	5.0	−0.8	2.5
OLSA 12 M (dB SNR)	77	−6.3	5.0	−1.4	2.2

Results of the Freiburg Monosyllabic Test (*FMS*, 65 dB SPL free-field presentation) and the Oldenburg Sentence Test in noise (*OLSA*, listwise case exclusion, OlNoise, fixed speech level 65 dB SPL, adaptive noise level, S0N0 presentation)

*SD* standard deviation

### Age at implantation and duration of hearing loss

The cohort’s mean age was 58.6 years (SD = 16.0), ranging from 20 to 92 years (Table 1). The self-reported duration of hearing loss was categorized. Hearing loss predominantly developed peri- or postlingually (*n* = 199), whereas congenital hearing loss was present in 54 cases, and no information on the duration of hearing impairment was available for 73 cases. The largest subgroup consisted of patients who acquired postlingual hearing loss before the age of 20 (*n* = 91).

### Mode of implantation

The classification of the mode of implantation was based on the description of test conditions recorded in the comment section of the free-field speech audiogram following CI fitting. The mode of implantation was categorized into five groups. *Unilateral *fitting referred to cases with exclusive unilateral CI use, as the contralateral ear could not be successfully fitted with a hearing aid due to functional deafness. In cases of asymmetric hearing loss (AHL), a CI was provided in one ear, while the contralateral ear was fitted with a well-functioning hearing aid (speech recognition score with a hearing aid in the FMS > 50%). The *bimodal* group also included CI users with hearing aid support in the contralateral ear, but with more limited benefit from the hearing aid (FMS score with a hearing aid ≤ 50%). Bilateral CI users were assigned to the *bilateral* category, with speech perception assessed separately for each ear. For further analysis, the CI ear with the better FMS score was selected and included in the dataset. Cases in which the contralateral ear had normal hearing were classified as single-sided deafness (SSD). Additionally, for all groups except AHL, some cases included electric-acoustic stimulation (EAS), which were assigned to their respective categories. The final distribution within the categories was as follows: unilateral (*n* = 22), AHL (*n* = 38), bimodal (*n* = 87), bilateral (*n* = 120), and SSD (*n* = 59).

### Kindergarten, school education and graduation, professional career

Educational background, including early childhood care, schooling, and professional qualifications, was assessed using a questionnaire (“Work and Life” form; ENT-Statistics database, Innoforce, Liechtenstein, digital supplement). The collected data included information on early childhood care, type of school attended, and educational and professional achievements. Early childhood care was categorized into four groups: *no kindergarten attendance, regular kindergarten, kindergarten for children with hearing impairments*, and *integrative kindergarten*. The distinction between the last two was that in the kindergarten for hearing-impaired children, only children with hearing loss were enrolled, whereas integrative kindergartens included both children with hearing impairments and those with normal hearing.

The classification of school types attended by patients was based on the level of specialized support provided. Cases in which no special educational support for individuals with hearing loss was received were assigned to the *regular school* category. Patients who attended schools exclusively for deaf students were grouped under the category of *school for the deaf*. Other cases in which students received any form of special support were categorized as *other* including schools for the hearing impaired, institutions for learning support, and integrative schools.

The study also classified patients based on their highest level of education and vocational training. School-leaving qualifications were categorized into *lower secondary school graduation, intermediate secondary school graduation, A‑levels* (including general and subject-specific higher education entrance qualifications), and cases with the absence of a school-leaving certificate (*no school-leaving certificate)*. Vocational qualifications were grouped into three categories: *no or miscellaneous vocational qualification, vocational school qualification*, and *higher professional qualification*. The last group included the education of master craftsman or technician training as well as degrees from universities of applied sciences and universities.

### Speech audiometry

Speech perception in quiet conditions was assessed using the FMS [15] at a presentation level of 65 dB SPL in a free-field setup. Testing was conducted in acoustically isolated audiometry rooms with a speaker positioned at a distance of 1 m from the participant. Data collection included results from preoperative assessments (with hearing aid use) as well as postoperative evaluations at 6 and 12 months after CI fitting. The tests were performed with the everyday settings of the participants’ CI processors. To assess speech intelligibility in noise, the OLSA [24] was used with a fixed speech level of 65 dB SPL and an adaptive noise level, with both speech and noise signals presented from the front (*S0N0 *condition). In participants with single-sided deafness and functional hearing in the contralateral ear, the non-implanted ear was occluded using earplugs and additional earmuff protection (Peltor) to prevent auditory input. The aim of the OLSA was to determine the speech reception threshold (SRT), defined as the SNR at which the participant correctly recognized 50% of the presented sentences. The test result was recorded in dB SNR, where a higher (positive) value indicated poorer speech comprehension, as it reflected a higher speech level relative to the noise level at the SRT.

### Statistical analysis

The statistical analysis was performed using SPSS Version 28.0 (IBM Corp., USA). Demographic data were analyzed by calculating means and standard deviations. Since the metric variables did not follow a normal distribution, as determined by the Shapiro–Wilk test, potential correlations between these variables and the outcome measures (FMS and OLSA) were assessed using Spearman’s rank correlation coefficient. The relationship between nominally scaled variables and the outcome measures was analyzed using ANOVA. If the assumption of homogeneity of variances (tested with Levene’s test) was violated, the Welch–ANOVA was applied. To control for potential confounding variables, an ANCOVA was subsequently performed. Effect sizes and the proportion of variance explained by ANOVA/ANCOVA were reported using eta-squared or partial eta-squared. Post hoc comparisons were conducted using the Bonferroni test in cases of homogeneous variances, whereas the Games–Howell test was applied when variance homogeneity was not met.

## Results

### Correlation and variance analysis

The results of the variance and correlation analysis were examined using the Spearman rank correlation coefficient (Table 2). A detailed evaluation at the processor and electrode level was not conducted, as the large variety of different device types resulted in small sample sizes, making meaningful conclusions difficult.

**Table 2:** Correlation of outcome variables with metric and categorical variables using the Spearman rank correlation coefficient and variance analysis (ANOVA)

	Spearman ρ *r*_*S*_ *| p*			
Metric variables	FMS 6 M†	FMS 12 M†	OLSA 6 M††	OLSA 12 M††
Age at implantation	−0.09 | 0.17	*−0.26 |<* *0.001*	0.21 | 0.07	*0.25 | 0.03*
Categorical variables	ANOVA Sig.			
Duration of hearing loss	0.10	0.19	0.33	0.20
Mode of implantation	*<* *0.001*	*<* *0.001*	0.07	*<* *0.01*
*Level of education*				
Type of Kindergarten	0.42	0.12	0.38	0.29
Type of school	0.07	0.05	0.59	0.43
Level of school-leaving graduation	0.32	0.37	0.17	0.14
Vocational Qualifications	*<* *0.01*	*0.05*	0.10	*0.02*

*n* (†) = 226, *n* (††) = 77, Freiburg Monosyllabic Test (*FMS*, 65 dB SPL free-field presentation) and Oldenburg Sentence Test in noise (*OLSA*, listwise case exclusion, OlNoise, fixed speech level 65 dB SPL, noise level adaptive, S0N0 presentation). Significant results highlighted

### Age at implantation and duration of hearing loss

A significant correlation was found between age at implantation and the FMS results 12 months postoperatively: *r*_*S*_ (224) = −0.26; *p* < 0.001. The negative sign of the correlation coefficient indicated that increasing age was associated with a decline in FMS performance. A significant correlation was also observed concerning the OLSA results 12 months postoperatively—*r*_*S*_ (75) = 0.25; *p* = 0.03—where higher age was associated with an increase in the SRT value, as indicated by a positive correlation coefficient. However, the duration of hearing impairment showed no significant correlation with speech perception test outcomes (Table 2).

### Mode of implantation

**Fig. 1:**
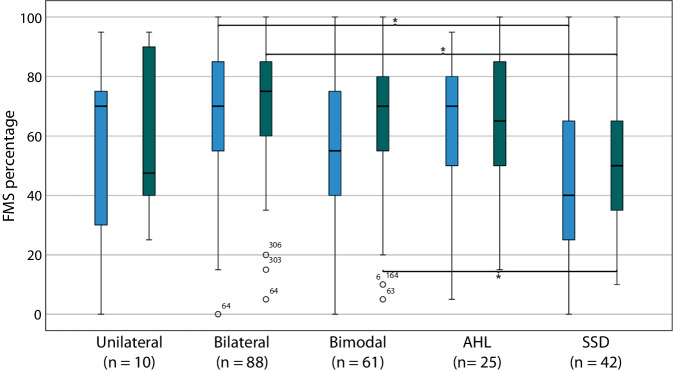
Boxplot Freiburg Monosyllabic Test (*FMS*) 6 M (*blue*) and 12 M postoperatively (*green*) in relation to mode of implantation, *n* = 226, (better side in bilaterally supported patients).*p < 0.05; FMS (65 dB SPL free-field presentation). *AHL* asymmetric hearing loss

Significant differences were observed between the different modes of implantation regarding FMS performance at both 6 months—*F* (4, 221) = 5.82; *p* < 0.001; η^2^ = 0.10—and 12 months—*F* (4, 221) = 6.95; *p* < 0.001; η ^2^ = 0.11—postoperatively. By contrast, significant differences in OLSA results were only observed at the 12-month test interval: *F* (4, 72) = 4.10; *p* < 0.01; *η *^*2*^ = 0.18. Post hoc analysis revealed that cases with SSD exhibited poorer FMS performance 6 months as well as 12 months postoperatively compared to those with bilateral implantation (*p* < 0.001; Table 3). After 12 months, SSD cases also showed worse performance in the FMS compared to bimodally fitted cases (*p* = 0.03; Fig. 1). Regarding OLSA outcomes (not shown), a significant negative association was found 12 months postoperatively between SSD and the following groups: bilateral (*p* < 0.01), bimodal (*p* = 0.03), and AHL (*p* = 0.04). Thus, SSD cases demonstrated better speech perception in noise compared to the other groups.

**Table 3:** Significant post hoc comparisons after Bonferroni correction

Dependent variable	Test variable	(I) Modes	(J) Modes	Mean difference (I-J)	SE	*p*
FMS 6 M	Mode of implantation	SSD	Bilateral	−20.7	4.7	*<* *0.001*
*	Vocational qualification	Vocational school qualification	Higher professional qualification	−12.4	3.7	*<* *0.01*
FMS 12 M	Mode of Implantation	SSD	Bilateral	−21.4	4.1	*<* *0.001*
		SSD	Bimodal	−13.3	4.4	*0.03*
	Vocational qualification	Vocational school qualification	Higher professional qualification	−8.3	3.4	*0.04*
OLSA 12 M	Mode of implantation	SSD	Bilateral	−2.5	0.7	*<* *0.01*
		SSD	Bimodal	−2.1	0.7	*0.03*
		SSD	AHL	−2.4	0.8	*0.04*
	Vocational qualification	No or miscellaneous vocational qualification	Vocational school qualification	2.1	0.7	*0.02*
		No or miscellaneous vocational qualification	Higher professional qualification	1.8	0.7	*0.04*

*n* (FMS) = 226, *n* (OLSA) = 77, Freiburg Monosyllabic Test (*FMS,* 65 dB SPL free-field presentation) and Oldenburg Sentence Test in noise (*OLSA*, listwise case exclusion, OlNoise, fixed speech level 65 dB SPL, noise level adaptive, S0N0 presentation). *SE* standard error, *SSD* single-sided deafness

* = Post hoc test according to Games–Howell, as variance homogeneity was not present

### Kindergarten, school education and graduation, professional career

For the majority of patients, the educational journey began with attendance at a regular kindergarten (*n* = 200), while 25 received specialized support, either in the form of a kindergarten for the hearing impaired (*n* = 19) or an integrative kindergarten (*n* = 6). Overall, 101 did not attend a kindergarten.

**Fig. 2:**
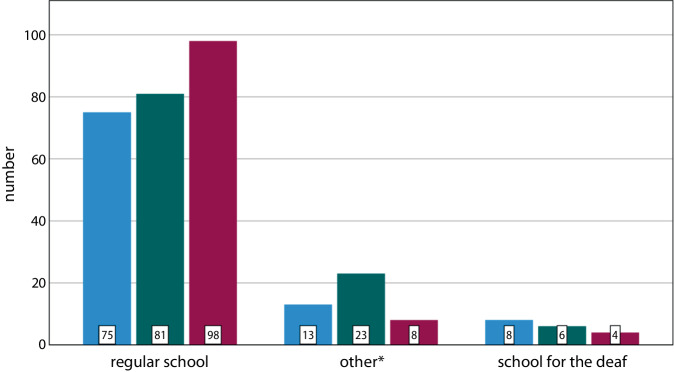
Bar chart of school graduation by school type. *Blue* = lower secondary school graduation, *green* = intermediate secondary school graduation, *red* = A-levels; total *n* = 316, with 10 cases without a school-leaving qualification. *Institutions for learning support (*n* = 6), integrative schools (*n* = 6), and schools for the hearing impaired (*n* = 36)

Most patients attended a regular school (*n* = 260). The majority obtained A‑levels (*n* = 98), followed by an intermediate secondary school graduation (*n* = 81) and a lower secondary school graduation (*n* = 75). Six patients completed their schooling without a formal qualification (Fig. 2). Regardless of the type of school attended, 110 patients obtained their A‑levels, 110 an intermediate secondary school graduation, 96 a lower secondary school graduation, and 10 did not receive any qualification. The category of A‑levels also included the subject-specific higher education entrance qualifications (*n* = 38) and the completion of the polytechnic upper level (*n* = 7). The category of *other* schools included institutions for learning support (*n* = 6), integrative schools (*n* = 6), and schools for the hearing impaired (*n* = 36).

**Fig. 3:**
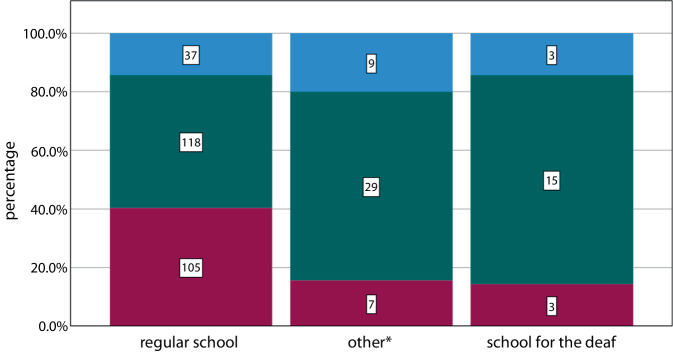
Bar chart of vocational qualification by school type. *Blue* = no qualification/other, *green* = vocational school qualification, *red* = higher professional qualification. Total number *n* = 326, *Institutions for learning support (*n* = 6), integrative schools (*n* = 6), and schools for the hearing impaired (*n* = 36)

After leaving school, the majority of patients pursued vocational training (*n* = 162), while some attended a master craftsman or technician school (*n* = 43) or obtained a university or university of applied sciences degree (*n* = 72). A total of 49 patients did not obtain a vocational qualification or they acquired a qualification of a different nature (Fig. 3).

Regarding educational attainment, neither the type of kindergarten attended— FMS 12 M: *F* (3, 222) = 1.97; *p* = 0.12, OLSA 12 M: *F*(3, 73) = 1.28; *p* = 0.29 —nor the level of school-leaving qualification obtained—FMS 12 M: *F* (3, 222) = 1.06; *p* = 0.37, OLSA 12 M: *F* (3, 73) = 1.86; *p* = 0.14—had a significant impact on speech perception outcomes in quiet or noise conditions. However, regarding the variable “type of school attended,” there was a trend 12 months postoperatively suggesting that patients who attended a school for the deaf exhibited slightly poorer speech comprehension in quiet conditions—FMS 12 M: *F* (2, 223) = 3.01; *p* = 0.05—compared to those who attended a regular school (*p* = 0.08) or another type of school (*p* = 0.06).

**Fig. 4:**
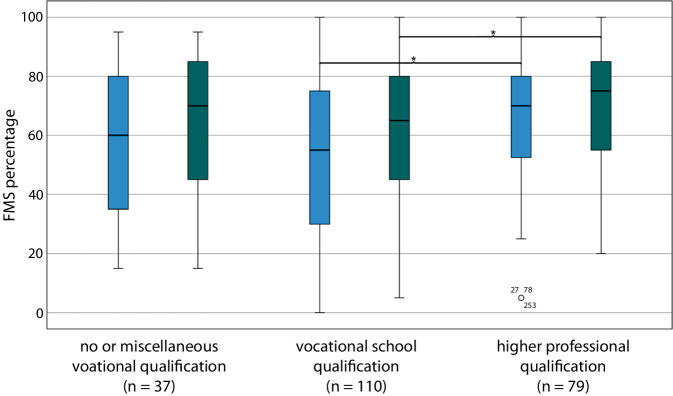
Boxplot Freiburg Monosyllabic Test (*FMS*) results 6 M (*blue*) and 12 M (*green*) postoperatively in relation to vocational qualification, *n* = 226. *p < 0.05; FMS (65 dB SPL free-field presentation)

For the variable “vocational qualification,” a significant group difference was observed: FMS 6 M: ANOVA_Welch_ *F*(2, 104) = 5.60; *p* = 0.05, FMS 12 M: *F*(2, 223) = 3.07; *p* = 0.05; *η *^*2*^ = 0.03. Patients with a vocational school qualification achieved significantly lower FMS test results compared to those with a higher professional qualification, both at 6 months (*p* < 0.01) and 12 months postoperatively (*p* = 0.04; Fig. 4). Even after adjusting for the covariates “age at implantation,” “duration of hearing impairment,” and “mode of implantation,” speech perception remained significantly associated with vocational qualifications at both 6 and 12 months postoperatively: ANCOVA FMS 6 M: *F*(2, 220) = 5.99; *p* < 0.01; partialη^2^ = 0.05, ANCOVA FMS 12 M: *F*(2, 220) = 4.05; *p* = 0.02; partialη^2^ = 0.03.

**Fig. 5:**
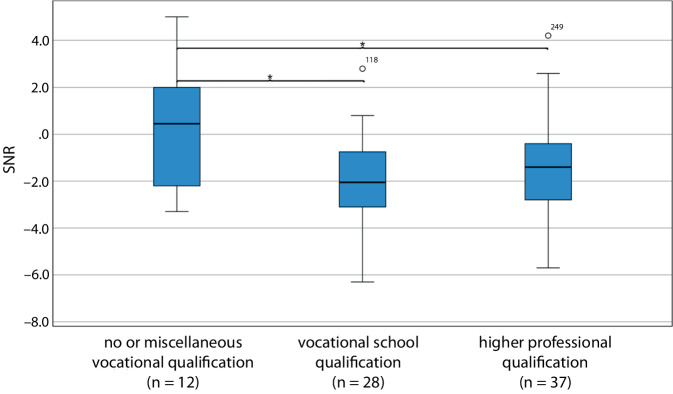
Boxplot of OLSA 12 M postoperatively in relation to vocational qualification, *n* = 77. *SNR* signal-to-noise-ratio. *p < 0.05. Oldenburg Sentence Test in noise (*OLSA*, listwise case exclusion, OlNoise, fixed speech level 65 dB SPL, adaptive noise level, S0N0 presentation)

For speech perception in noise at 12 months postoperatively, significant group differences were also observed: OLSA 12 M: *F* (2, 74) = 4.41; *p* = 0.02;η^2^ = 0.11. Considering the covariates “age at implantation,” “duration of hearing impairment,” and “mode of implantation,” a significant group difference remained for the variable “vocational qualification” in terms of speech perception in noise: ANCOVA OLSA 12 M: *F* (2, 71) = 3.96; *p* = 0.02; partialη^2^ = 0.10. Post hoc analysis revealed poorer performance in patients with no or miscellaneous qualification compared to those with a vocational school qualification (*p* = 0.02) or a higher qualification (*p* = 0.04; Fig. 5).

## Discussion

### Summary of results

The primary aim of the present study was to investigate the influence of education level on hearing performance after CI fitting. Significant associations were found between the level of vocational education and speech perception in both quiet and noisy environments. Patients with a vocational school qualification achieved significantly poorer results than those with a higher professional education, both at 6 months postoperatively (*p* < 0.01) and at 12 months postoperatively (FMS, *p* = 0.04; Fig. 4). In noise, the group with no or miscellaneous qualification performed worse at 12 months postoperatively compared to those with a vocational school qualification (*p* = 0.02) or higher professional qualification (*p* = 0.04; Fig. 5). Other educational parameters, such as early childhood care, school type, or school-leaving certificate, showed no significant associations with speech perception.

### Educational attainment and cognition

Educational attainment was considered a potential indicator of cognitive performance in this study. The influence of cognition on CI outcomes has been extensively described in the literature. Several authors have demonstrated positive correlations between verbal learning ability and auditory working memory with speech perception [16, 20, 35]. This effect has also been observed in CI-treated children, as reported by Pisoni [34]. Additionally, visual memory performance and the ability to make nonverbal inferences correlate with numerical comprehension but not with spoken words or sentences [23]. The present study reveals a weak correlation between educational attainment, as a possible indicator of cognitive ability, and speech perception in CI patients. However, this variable accounts for only a small percentage of the overall variance in speech perception test results. It is possible that educational attainment is not a reliable indicator of individual intelligence and cognition, as previously suggested by Heutink [18]. Heutink and colleagues classified 129 patients according to the Dutch education system based on their highest educational qualification. A Bachelor of Science (BSc) degree or higher was defined as “high educational attainment” and was achieved by 23 study participants. The Mann–Whitney test showed no correlation between educational attainment and CI speech perception outcomes. This result may be attributed to the small sample sizes in the statistical analysis. By contrast, the smallest group in the present study comprised 49 cases (no or miscellaneous qualification), while a high level of education was achieved by 115 individuals, ensuring greater statistical power in group comparisons. Significant differences may only be measurable in larger groups. Another study by Lazard et al. defined educational attainment based on the duration of schooling or vocational training [25]. The authors found no significant association between educational attainment and patients’ speech perception outcomes (*p* = 0.25). Although Lazard et al. had larger sample sizes than Heutink et al., they did not differentiate by type of education but only by the total years spent in schooling or vocational training (group 1: < 12 years, *n* = 21; group 2: 12–18 years, *n* = 501; group 3: > 18 years, *n* = 518, no information: *n* = 1211). This approach allows for only an arbitrary assessment of educational quality, and the groups may be too homogeneous to detect statistically significant differences.

In the present study, educational attainment was primarily classified based on school type and final qualification. However, even within the same school type, regional differences in the quality and quantity of education may exist. These differences are difficult to measure and could introduce bias into the study results. Additionally, educational attainment should be regarded only as a potential indicator of cognitive ability. Other factors may also influence patients’ educational pathways. For instance, prioritizing an alternative lifestyle unrelated to higher education does not necessarily imply lower cognitive ability. Social and health-related circumstances may also shape educational trajectories, leading to early completion or discontinuation of school or vocational education. More precise insights could be obtained through cognitive testing.

A portion of the data presented in this study was analyzed in an abstract submitted to the 26th Annual Meeting of the German Society for Audiology (DGA) [4]. Due to a slightly different grouping of the “school type” variable (combining cases from schools for the hearing impaired and deaf schools), the authors demonstrated that school type, in addition to vocational qualification, was significantly associated with CI speech perception outcomes. This finding supports the statistical trend observed in the present study regarding the influence of school type on speech perception. Overall, the data suggest that patients with higher educational attainment achieve better results in speech perception tests. However, since educational attainment explains only a very small portion of the variance in speech perception outcomes, the clinical significance of this finding may be limited.

### Impact of hearing aid use on socioeconomic position

The impact of educational attainment on hearing aid use is well documented in the literature [17, 31, 38]. Helvik et al. reported that, among 11,602 patients aged ≥ 65 years, hearing aid use was positively correlated with higher educational attainment. Malcolm et al. further described that not only educational attainment but also overall socioeconomic position is associated with hearing aid use. Patients with a lower socioeconomic position were less likely to use hearing aids, were more frequently unemployed, and had lower incomes. Overall, there is a clear positive correlation between hearing aid use and educational attainment. However, this association is difficult to directly relate to the present study, as the focus here is not on CI utilization but on the effectiveness of CI treatment (measured by speech perception) in relation to educational attainment.

### Age at implantation

Age at implantation is a well-established factor influencing speech perception after CI, as demonstrated in numerous studies [3, 10, 14, 29, 37]. The interaction between cognition and speech perception has also been described in the literature. In a study by Holden et al., the authors found that after adjusting for age, no significant correlations remained between cognition and speech perception [20]. It is well documented that age-related changes in hearing occur in both central and peripheral auditory structures [13]. Similarly, age-related cognitive decline is a well-established phenomenon [37]. Both factors may contribute to the observed decline in hearing performance in older CI patients. In the present study, a negative correlation was found between age at implantation and speech perception in quiet, while a positive correlation was observed between age and speech perception in noise at 12 months postoperatively. However, some studies have found no age-related effects. Leung et al. showed that in a cohort of CI patients aged ≥ 65 years, implantation age was not a significant factor [28]. Under the same conditions (implantation at ≥ 65 years), no significant correlation with speech perception was found in the present study, neither in quiet—FMS 6 M: *r*_*S*_ (57) = −0.23; *p* = 0.08, FMS 12 M: *r*_*S*_ (57) = −0.13; *p* = 0.32—nor in noise— OLSA 6 M: *r*_*S*_ (13) = −0.10; *p* = 0.75, OLSA 12 M: *r*_*S*_ (13) = 0.12; *p* = 0.69. Budenz et al. noted that when the duration of hearing impairment was taken into account, age at implantation was no longer significantly correlated with speech perception [5]. Overall, the results of the present study confirm that age at implantation is a significant influencing factor on speech perception in CI patients when a broader age distribution is considered.

### Duration of hearing impairment

The duration of hearing impairment is arguably the strongest and most well-documented factor influencing speech perception after CI [1, 3, 11, 20]. The longer the hearing impairment persists, the poorer CI performance appears to be in terms of speech perception. In particular, the duration of severe to profound hearing loss (SPHL) is a decisive factor. In the present study, no significant correlation was found between the duration of hearing impairment and speech perception after CI. This could be due to self-reported patient history data, which may be affected by recall bias. Determining the exact onset of hearing loss is challenging, and therefore patients were categorized into broad groups (*congenital, perilingual, postlingual <* *5 years, postlingual 5–20 years, postlingual >* *20 years*). This classification may have masked existing differences. A systematic review by Bernhard et al. analyzing 36 studies confirmed that overall, the duration of hearing impairment negatively correlates with speech perception in CI patients [1]. Longer hearing impairment duration leads to poorer outcomes. However, this factor becomes less significant with increasing CI experience. While the duration of hearing loss has been described as an important factor in the literature, it was not significantly associated with speech perception in the present study.

### Mode of implantation

Regarding speech perception in quiet (FMS), the results of this study suggest poorer speech perception in single-sided deafness (SSD) patients. Speech perception in noise (OLSA) produced mixed results. These differences may be related to the protocol of masking applied for tests with SSD patients. During the OLSA test, for patients with unilateral deafness and usable hearing in the contralateral ear, the non-implanted ear was masked using earplugs and a Peltor earmuff (3M Peltor Optime II). This masking method may not have fully excluded the non-tested ear, potentially leading to better results for SSD patients due to their normal hearing on the contralateral side. Another source of uncertainty arises from the classification of the mode of implantation, which was determined preoperatively based on free-field speech audiometry with hearing aids. In some cases, it may have changed postoperatively without being recorded in the database. Therefore, further studies are necessary to verify the impact of “mode of implantation” on speech perception outcomes.

### Limitations

In addition to the limitations already discussed in the Discussion section, there are potentially other factors that may influence the results of the study: Information regarding the individuals’ hearing loss history was typically obtained through a self-reported anamnesis. In addition, particularly in older patients, inaccurate information regarding the educational system may have been provided due to inaccurate memory. Furthermore, the presence of various implant systems, electrode designs, and CI processors complicates the comparison of test results, as technological differences may affect hearing performance. The follow-up periods for the FMS and OLSA were 6 and 12 months postoperatively. A relatively broad time window of ± 4 months was used for the evaluation, which could make it difficult to compare patient outcomes. In the current study, several other influencing factors were considered, some of which had a significant impact on speech perception (age at implantation, mode of implantation). The results were adjusted for these covariates, but other unidentified covariates may obscure the influence of the level of education on speech comprehension.

### Summary

The data from this study confirmed several well-known factors influencing CI outcomes. Overall, the findings suggest that patients’ educational level may impact speech perception after CI fitting. A more detailed classification of different educational levels with larger datasets could help validate this relationship.

## Practical conclusion

- Educational level shows a weak correlation with speech perception after cochlear implantation (CI).
- A higher educational level appears to have a slight positive effect on speech perception.
- A significant portion of the variance in speech perception after CI remains unexplained.

## Supplementary Information

Work and Life form, ENT-Statistics database, Innoforce, Liechtenstein

## Data Availability

The data that support the findings of this study are not openly available due to reasons of sensitivity and are available from the corresponding author upon reasonable request. Data are located in controlled access data storage at Goethe University Frankfurt, Germany.

## Declarations

All human studies or investigations involving human tissue described in this research were conducted in accordance with national regulations and the Declaration of Helsinki (1975, in its current revised version). Ethical approval for this study was obtained from the Ethics Committee of the Faculty of Medicine at J.W. Goethe University Frankfurt am Main (Approval Number: 2022-642). Informed consent was obtained from all participating patients.

The supplement containing this article is not sponsored by industry.
